# Use of Periodic Mesoporous Organosilica–Benzene Adsorbent for CO_2_ Capture to Reduce the Greenhouse Effect

**DOI:** 10.3390/ma17112669

**Published:** 2024-06-01

**Authors:** David Cantador-Fernandez, Dolores Esquivel, José Ramón Jiménez, José María Fernández-Rodríguez

**Affiliations:** 1 Departamento de Química Inorgánica e Ingeniería Química, Campus de Rabanales, Edificio Marie Curie, Universidad de Córdoba, 14071 Córdoba, Spain; p12cafed@uco.es; 2 Departamento de Química Orgánica, Universidad de Córdoba, 14001 Córdoba, Spain; q12esmem@uco.es; 3 Instituto para la Energía y el Medioambiente (IQUEMA), Universidad de Córdoba, 14071 Córdoba, Spain; 4 Departamento de Ingeniería Rural, Escuela Politécnica Superior de Belmez, Universidad de Córdoba, Ed. Leonardo Da Vinci, Campus de Rabanales, Ctra. N-IV, km-396, 14001 Córdoba, Spain

**Keywords:** CO_2_ capture, CO_2_ isotherms, PMO–benzene, loss of CO_2_ capture capacity, climate change

## Abstract

The CO_2_ adsorption of a phenylene-bridged ordered mesoporous organosilica (PMO–benzene) was analyzed. The maximum capture capacity was 638.2 mg·g^−1^ (0 °C and 34 atm). Approximately 0.43 g would be enough to reduce the amount of atmospheric CO_2_ in 1 m^3^ to pre-industrial levels. The CO_2_ adsorption data were analyzed using several isotherm models, including Langmuir, Freundlich, Sips, Toth, Dubinin–Radushkevich, and Temkin models. This study confirmed the capability of this material for use in reversible CO_2_ capture with a minimal loss of capacity (around 1%) after 10 capture cycles. Various techniques were employed to characterize this material. The findings from this study can help mitigate the greenhouse effect caused by CO_2_.

## 1. Introduction

Excessive increases in CO_2_ concentration over a short period of time lead to an amplification of the greenhouse effect and a sharp rise in the average temperature of the planet. Before the industrial revolution, CO_2_ levels were 280 ppm [1]. As a result of human activities, a CO_2_ level of 421 ppm was reached in 2022 [2,3,4]. To address this situation, many international organizations and governments have worked to reach common agreements. One of the most important agreements was the Paris Agreement (2016) [5]. The capture of CO_2_ [6,7] could allow its use as a revalorized by-product, such as fuels, chemicals, and building materials, adding an economic incentive for CO_2_ capture and the green economy, while also reducing the environmental footprint [8,9]. Different adsorbents such as the diamine-based hybrid-slurry system [10], porous carbons [11,12], silica [13,14,15], MOFs [16,17,18], metal oxides [19,20,21,22], hydrotalcites [23,24], and periodic mesoporous organosilicas (PMOs) have been studied recently for CO_2_ capture.

PMO materials have a considerable interest for multiple applications [25] such as catalysis [26,27,28,29,30,31], metal adsorbents [32,33], organic pollutant adsorbents [34,35,36], gas adsorbents [37,38,39], chemosensors [40,41], optical devices [42], and the immobilization of enzymes or therapy in cancer cells [43,44].

Inagaki et al. [45], Melde et al. [46], and Asefa et al. [47] published the synthesis of a new homogeneously distributed mesoporous organic–inorganic material using bis(trimethoxysilyl)ethane, bis(triethoxysilyl)ethane, and bis(triethoxysilyl)ethene as precursors, respectively. This was the first synthesized PMO material. Subsequently, in 2002, Kuroki et al. [48] synthesized a new PMO material containing a benzene functional group for the first time using 1,3,5-Tris(triethoxysilyl)benzene and 1,3-Bis(triethoxysilyl)benzene as precursors and Cetylpyridinium chloride (CPCl) or cetyltrimethylammonium bromide (CTABr) as surfactants.

Selecting specific organosilica types and synthesis conditions enables precise control over the size, shape, uniformity, and periodicity of the pore structures [49]. However, the shape of the adsorbent particle determines the specific surface area.

Several authors have addressed CO_2_ adsorption using PMO materials [50,51,52,53,54,55,56,57] at low pressure and temperatures of 0 °C and 25 °C, with different results.

**Figure 1:**
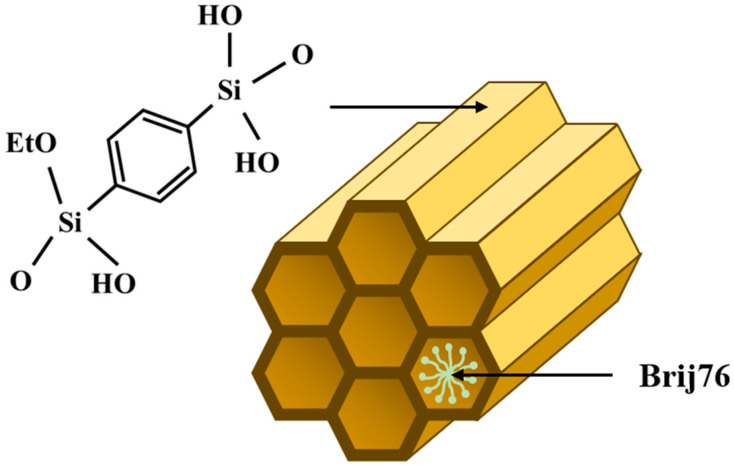
Representation of PMO–benzene structure.

In this study, a PMO with phenylene bridges was chosen as the CO_2_ adsorbent material (Figure 1). Various techniques were utilized to characterize this material, and its CO_2_ capture capacity at low temperatures (0 °C, 10 °C, 20 °C, and 35 °C) and high pressure (35 atm) was evaluated. The CO_2_ adsorption data were fitted by the Langmuir, Freundlich, Sips, Toth, Dubinin–Radushkevich, and Temkin models.

The capability of this material for use in reversible CO_2_ capture with a minimal loss of capacity after 10 capture cycles was addressed.

The obtained results can aid in the reduction in the CO_2_ greenhouse effect.

## 2. Materials and Methods

### 2.1. PMO–Benzene

Periodic mesoporous organosilica (PMO–benzene) was synthesized via acid-catalyzed hydrolysis and the condensation of bis(triethoxysilyl)benzene following the method outlined by Burleigh [58,59]. In this process, 6 g of the surfactant BrijS10-Brij76 (Polyoxyethylene (10) stearyl ether) was dissolved in a solution of 19.6 mL of HCl and 279 mL of H_2_O while being stirred at 50 °C for 24 h. Subsequently, 17.2 mL of 1,4-bis(triethoxysilyl)benzene was added, and the mixture was continuously stirred at 50 °C for another 24 h. The solution was then kept at 90 °C under static conditions for an additional 24 h. The resulting precipitate was collected through vacuum filtration and washed with H_2_O. To remove the surfactant, the synthesized material was stirred in a HCl solution (1:50 ratio) at 80 °C for 12 h. Finally, the product was filtered, washed with ethanol, and dried under vacuum for 10 h. The molar ratios of Brij76, H_2_O, HCl, and organosilane used in the reaction were 0.11:222:3.20:0.56.

### 2.2. Material Characterisation

Different techniques were used for the characterization of the PMO–benzene samples. XRD analysis was conducted using a Bruker D8 Discover A25 instrument (Karlsruhe, Germany), utilizing the ICDD 2003 database [60]. N_2_ adsorption isotherms were used to evaluate S_BET_ and porosity, conducted with the Autosorb iQ2 from Quantachrome Instruments (Boynton Beach, FL, USA). Prior to this, the samples underwent degassing for 24 h at 70 °C. The data were processed with AsiQwin software (version 3.0). The surface area was calculated using the Brunauer–Emmett–Teller (BET) method. Density functional theory (DFT) was used to test the porosity. The pore volumes were calculated on the basis of the single-point method. A Mastersizer S laser diffractometer (Malvern, UK) was used to determine the particle size. Particle aggregates were separated during an ultrasonic bath (10 min). Particle structure was analyzed using a transmission electron microscope (Talos F200i TEM; Thermo Fisher Scientific) (Waltham, MA, USA). A Sievert PCTPro-2000 instrument (Setaram) (Caluire-et-Cuire, Francia) was used to obtain the CO_2_ isotherms. The gases CO_2_ 4.5 (99.995%) and He 5.0 (99.999%) were used.

### 2.3. Adsorption Isotherms

The CO_2_ isotherms for the PMO–benzene, measured at different temperatures (0 °C, 10 °C, 20 °C, and 35 °C), were fitted to several models [23,61]: Langmuir [62,63], Freundlich [64], Sips [65], Toth [66], Dubinin–Radushkevich (D-R) [67,68], and Temkin [69,70]. A detailed characterization was described by Cantador et al. [23]. A brief description is provided below.

Langmuir characterizes monolayer adsorption on a uniform surface [71] with identical adsorption sites, indicating that the energy remains constant regardless of the amount adsorbed [72]. Since adsorption takes place in a single layer, the surface area can be determined. In 1995, Tóth proposed [66] a correction factor χ_L_ to enhance the accuracy of monolayer adsorption calculations.

Freundlich, on the other hand, describes adsorption on heterogeneous surfaces [73,74], enabling the assessment of the surface’s heterogeneity and the adsorption intensity.

The Sips model integrates aspects of both the Langmuir and Freundlich models by depicting Freundlich-type adsorption at low pressures and resembling the Langmuir model at high pressures, thereby unifying the two approaches [75]. Analogously to the Langmuir model, a correction factor, χ_S_, was applied.

Toth’s model modifies Langmuir’s equation to accurately describe adsorption isotherms that include both monolayer and multilayer formations up to the maximum relative pressure.

The D-R model is employed to describe the CO_2_ adsorption based on potential energy during pore filling, which in turn determines adsorption capacity.

The Temkin model explains the heat generated in the pore filling process on the solid surface, establishing a correlation with the quantity of gas adsorbed.

MATLAB software version R2015a was used for calculation and fitting.

## 3. Results and Discussion

### 3.1. Characterization

**Figure 2:**
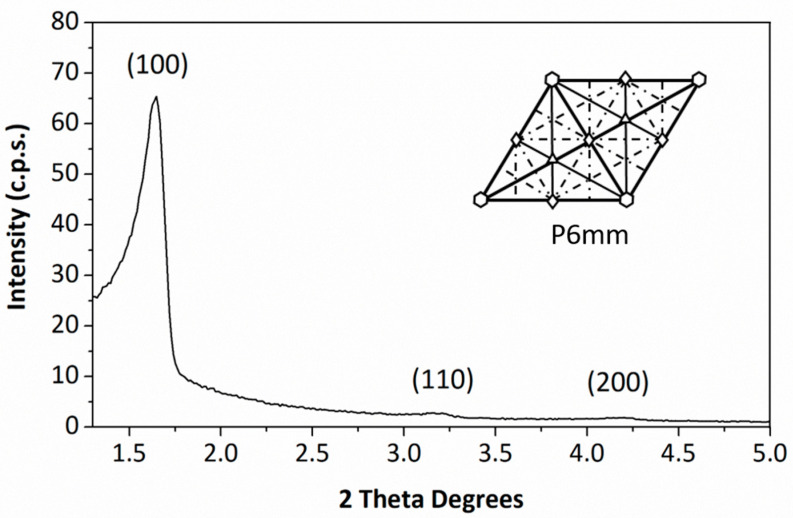
XRD patterns of PMO–benzene. The goniometer speed was 0.02°⋅s^−1^.

High-quality PMO–benzene was characterized. As illustrated in Figure 2, the XRD pattern of the sample displays its most prominent peak (100) at d = 53.4 Å. Additionally, two smaller and broader peaks were observed at approximately d = 27.3 Å (110) and d = 21 Å (200). These peaks confirm that the sample is a mesoporous material with a 2D hexagonal (P6mm) structure, characteristic of the organosilica PMO–benzene, consistent with the literature [36,76].

**Figure 3:**
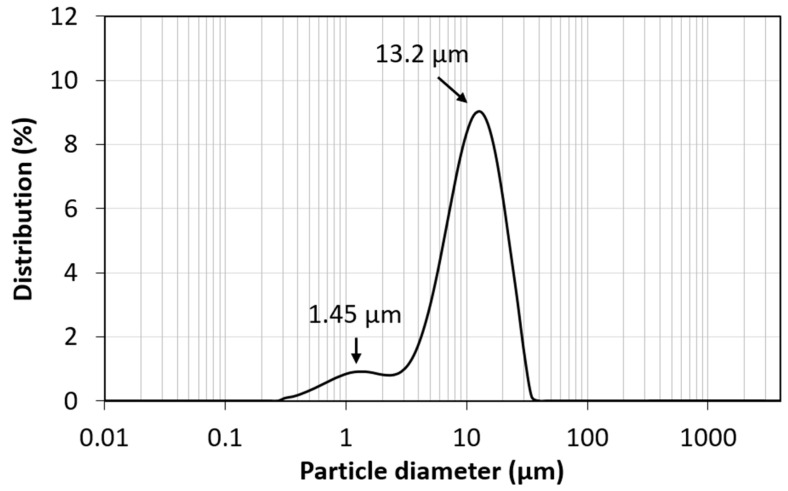
Particle size for PMO–benzene.

Figure 3 presents the particle size distribution, revealing a bimodal pattern. The primary peak is narrow, ranging from 2.5 to 35 μm and centered at 13.2 μm, suggesting uniformity in particle size within the sample. A secondary peak appears between 0.3 and 2.5 μm, with a center at 1.45 μm, indicating the presence of a smaller particle group.

**Figure 4:**
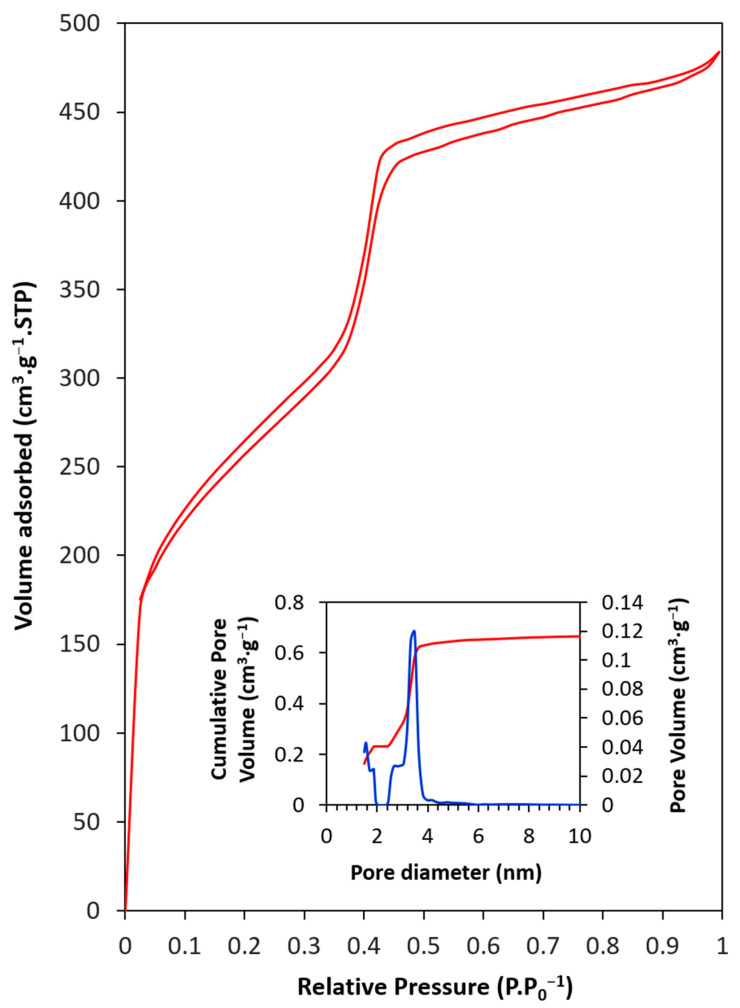
Nitrogen adsorption–desorption isotherms and pore size distribution of PMO–benzene. In the inserted figure: (Blue line) Pore volume, (Red line) Cumulative pore volume.

Figure 4 displays the N_2_ adsorption–desorption isotherm, which is Type IV according to the IUPAC. The first portion of the isotherm corresponds to monolayer–multilayer adsorption, with a well-defined adsorption limit at pressures approaching p·p_0_^−1^ = 1 [77,78]. The hysteresis in the multilayer region is indicative of capillary condensation within the mesopores. This hysteresis is classified as type H1, which is associated with a pore system that features a relatively regular arrangement and a narrow distribution of uniform mesopores [78,79], consistent with the XRD-derived crystal structure. Most pores were small mesopores, ranging from 2.4 to 4.1 nm, with a predominant pore diameter of 3.47 nm, and the total pore volume was 0.68 cm^3^·g^−1^ (Table 1). The BET method revealed a high surface area of 928 m^2^·g^−1^, which explains the material’s significant adsorption capacity. Additionally, several micropores were identified, contributing 139 m^2^·g^−1^ to the total surface area, representing 15% of the total.

**Figure 5:**
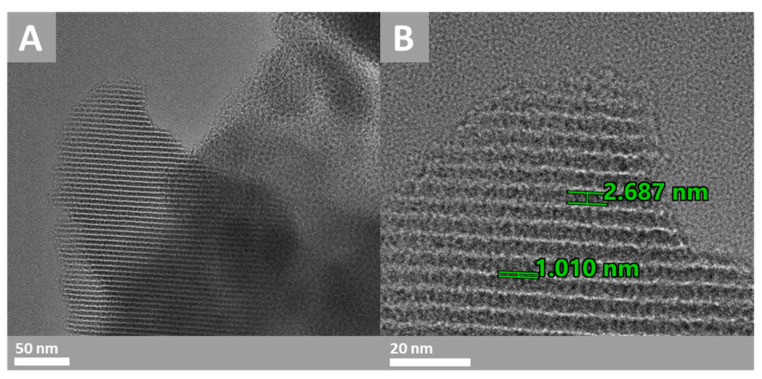
Images of PMO–benzene: (**A**) TEM and (**B**) high-resolution TEM.

A TEM was used on the PMO–benzene samples. Figure 5A shows the ordered pore structure of the material from two perspectives: longitudinal and transverse to the pore network. Figure 5B shows channels with a porous diameter around 2.867 nm, i.e., a pore size which agrees with the pore-size distribution (2.4–4.1 nm). The value of 1.01 nm corresponds to the thickness of the channel wall.

### 3.2. CO_2_ Adsorption

**Figure 6:**
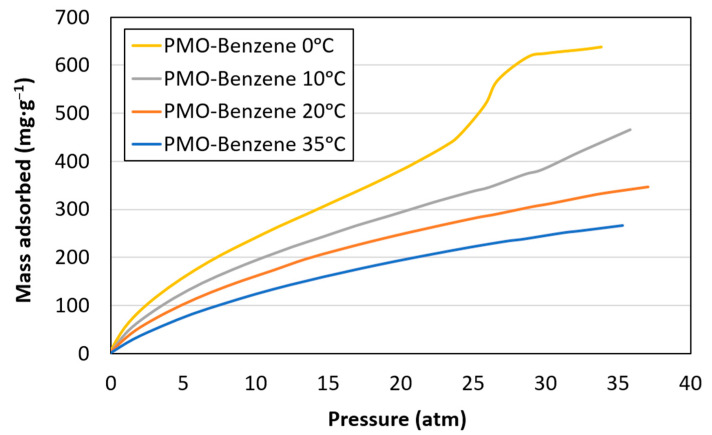
CO_2_ isotherms at 0 °C, 10 °C, 20 °C and 35 °C.

Figure 6 illustrates the CO_2_ adsorption capacity of PMO–benzene at various temperatures (0 °C, 10 °C, 20 °C, and 35 °C) and pressures ranging from 0 to 35 atm. The isotherm at 0 °C displayed a significant resemblance to the N_2_ isotherms, suggesting similar adsorption behavior. Monolayer formation was observed around 13.3 atm (p·p_0_^−1^ = 0.38), calculated using MATLAB R2015a, with multilayer formation extending to p·p_0_^−1^ = 1. For the samples at 10 °C and 20 °C, multilayer formation was noted at 20.6 atm (p·p_0_^−1^ = 0.46) and 30.7 atm (p·p_0_^−1^ = 0.54), respectively. At 35 °C, saturation pressure was not achieved, and monolayer filling was incomplete at 35 atm.

The CO_2_ adsorption capacity decreased as the temperature increased. The highest adsorption capacity was observed at 0 °C (638.2 mg·g^−1^), consistent with previous characterization results.

**Figure 7:**
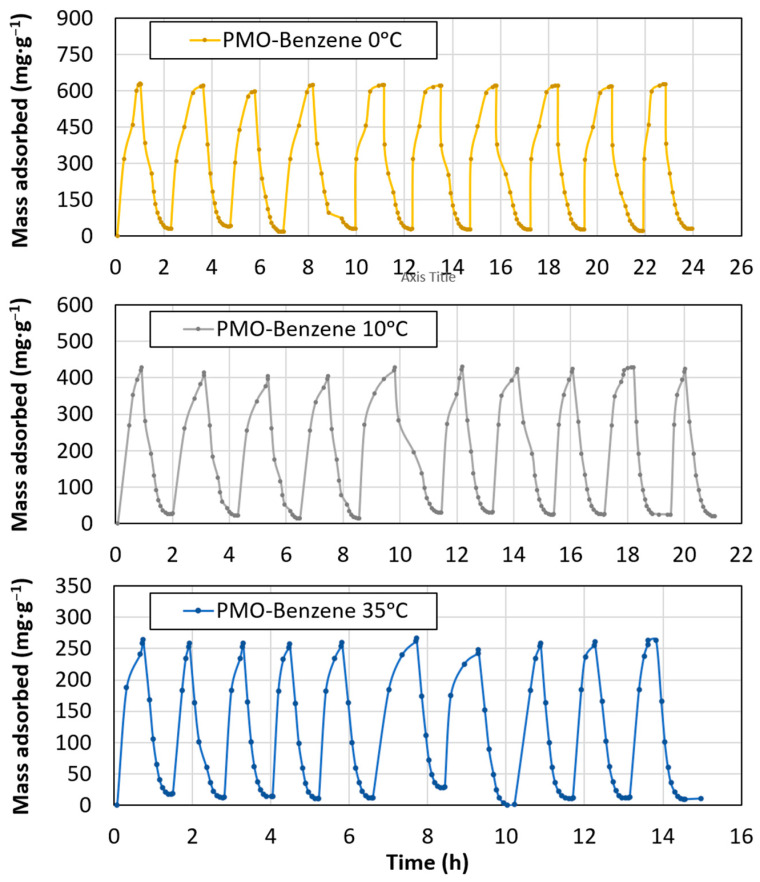
CO_2_ adsorption cycles at 0 °C, 10 °C, and 35 °C.

**Figure 8:**
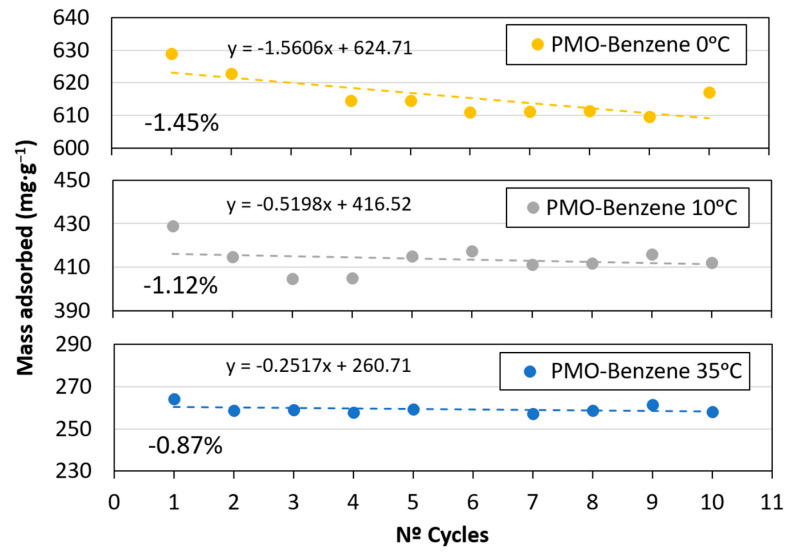
Loss of CO_2_ adsorption capacity after 10 cycles at 0 °C, 10 °C, and 35 °C.

In order to analyze the decrease in adsorption and to explore the potential for reusing the material as a CO_2_ adsorbent, a study was conducted involving 10 consecutive adsorption–desorption cycles at 0 °C, 10 °C, and 35 °C (Figure 7). The linear trend and maximum adsorption for each cycle are shown in Figure 8. After 10 cycles, there was a small loss in adsorption in all cases, with loss amounts between 1% and 1.5%. The working temperature did not seem to affect the adsorption capacity loss. The results indicate the material’s potential for reuse without significant loss of performance.

Various models were used to fit the adsorption data.

**Figure 9:**
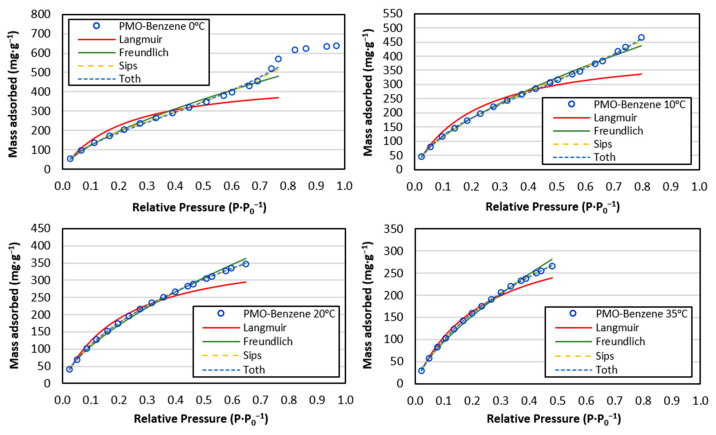
Fit curves of various models at 0 °C, 10 °C, 20 °C, and 35 °C for PMO–benzene.

All the isotherms fit the Freundlich model well (Figure 9). For the Langmuir model, only the isotherms at 0 and 10 °C obtained an R^2^ value below 0.99 due to multilayer formation and significant adsorption in the final stage (Table 2). The samples fit the Freundlich model slightly better than the Langmuir model. The Freundlich model provided a better fit, indicating surface heterogeneity (n), with results suggesting a somewhat heterogeneous surface.

According to Giles et al.’s [80] classification, the isotherms exhibited characteristics between L-type, indicative of strong intermolecular attractions and a gradual reduction in available adsorption sites, and C-type, where the number of adsorption sites remains constant. L-type curves typically align with Langmuir adsorption behavior, while C-type curves suggest linear adsorption. This shape is associated with weak intermolecular forces and a gradual occupation of adsorption sites. The monolayer filling values (qm) were generally overestimated, but using the Tóth correction yielded accurate monolayer filling predictions (qmc). This adjustment enabled the calculation of specific surface area using the Langmuir model. At 0 °C, where the adsorption curve reached a p·p_0_^−1^ of 1, the specific surface area (SL) was 904.1 m^2^·g^−1^, closely matching the BET method’s value of 928 m^2^·g^−1^ (Table 1). The Freundlich intensity factor (nf), which measures the interaction strength between the adsorbent and adsorbate at low pressures, was above 1 but close to it, indicating favorable, albeit weak, interactions [66]. This result agrees with Giles et al.’s [80] interpretation. The Langmuir equilibrium constant (K_L_) and Freundlich nf values (Table 2) increased with temperature, except at 35 °C, consistent with findings from other CO_2_ capture materials in the literature [23,24,61]. The Freundlich adsorption capacity per unit concentration (Kf) increased as the temperature decreased, directly correlating with higher CO_2_ adsorption capacity.

These models effectively showed the adsorption trends (qmc and Kf), which rose as the temperature decreased. The Freundlich model provided a more accurate prediction of the maximum adsorption capacity (Figure 9) compared to the Langmuir model, which only accounts for monolayer adsorption.

The Sips and Toth models (three-parameter) improved the fit over the Langmuir and Freundlich models for samples between 0 and 20 °C, reflecting in higher R^2^ values (Table 3). These models used relative pressures (p·p_0_^−1^) of 0.38, 0.46, and 0.54 as starting points for multilayer formation for samples at 0 °C, 10 °C, and 20 °C, respectively, obtained from the adsorption curve’s derivative. For the 35 °C sample, where no multilayer formation occurred, the maximum p·p_0_^−1^ value was used for fitting (Figure 9), though the parameter values are not listed in Table 3. The temperature-dependent trends for qs (Sips) and qT (Toth) differed from qmc (Langmuir) due to the models’ varying interpretations of the adsorption curve. The Langmuir model treated the curve as a monolayer over the entire pressure range, whereas the Sips and Toth models incorporated the pre-parameter for multilayer initiation, which varied with temperature. The Sips and Toth models predicted the CO_2_ adsorption capacity equally well, as indicated by the R^2^ values. The increasing qs and qt values with temperature are related to the increase in the relative pressure (p·p_0_^−1^) at which the multilayer is formed. This could be related to the increase in curvature at low p·p_0_^−1^ values and is in accordance with the nf value of the Freundlich model (the higher the initial curvature, the higher the nf). The heterogeneity factors from the Sips (n_S_) and Toth (n_T_) models agreed with the Freundlich model’s heterogeneity factor (n).

This research emphasizes the importance of using three-parameter models alongside classical two-parameter models to achieve better isotherm fitting, particularly when multilayer formation occurs (Figure 9).

For the D-R model, all isotherms had R^2^ values greater than 0.9. These adsorption capacities (q_D_) were slightly higher than those determined by the Langmuir model (qmc). The adsorption constant (β) was used to determine the free energy (E), which was smaller than 8 kJ⋅mol^−1^ for all samples (Table 4), suggesting the predominantly physical nature of adsorption. A slight increase in E was observed with increasing temperature.

A relationship between the D-R adsorption (E) and Temkin maximum binding energy (KTk) was observed, with both parameters increasing upon increasing temperature. The same trend was observed for the Temkin constant (bTk). These findings align with the expectation of low adsorption energy [81]. The physical nature of adsorption and the weak intermolecular attraction between adsorbent and adsorbate, as indicated by the fits, support the reversibility of CO_2_ capture. This is further evidenced by the minimal loss of adsorption after 10 cycles (Figure 7 and Figure 8). These properties suggest that the material is suitable for reversible multicycle CO_2_ capture by changing pressure conditions.

Table 5 provides a summary of significant studies on CO_2_ capture using mesoporous materials. Most of these studies focus on single temperature and pressure conditions and do not include adsorption–desorption cycling analyses. This research, however, examines the adsorption performance of PMO–benzene across various operating conditions.

Sim et al. [50] and Sim et al. [51] studied PMO–benzene samples functionalized with N-[3-(trimethoxysilyl)propyl] ethylene-diamine and PEO, respectively. The best result amounted to 133.32 mg·g^−1^ and was obtained for PMO–benzene (25 °C and 1 atm) modified with N-[3-(trimethoxysilyl)propyl] ethylene-diamine [50].

Other studies on PMOs at standard pressure and 0 °C have been conducted by Kirren et al. [52] and Wei et al. [53] (modified organosilica PMO–ethane), Liu et al. [54] (periodic mesoporous organosilica with a basic urea-derived framework), Rekha et al. [55] (PMO–cyclophosphazene), and Xu et al. [56] (polyethylenimine75-MCM-41) at standard pressure and 75 °C. In contrast, Lourenço et al. [57] worked at 10 atm. The maximum adsorption was 133 mg·g^−1^ for polyethylenimine75-MCM-41 at 75 °C and 1 atm [56].

The findings of this study indicate that CO_2_ adsorption at 34 atm pressure and temperatures ranging from 0 °C to 35 °C surpasses previously reported values for PMOs in the literature.

Table 5 compares these results with those for other mesoporous materials, such as those studied by Chowdhury et al. [20], Bhagiyalakshmi et al. [82], Wang et al. [83], Niu et al. [84], Liu et al. [21] and Li et al. [85]. The best result was 391.6 mg·g^−1^ (25 °C and 10 atm). In the current study, better results were obtained when working between temperatures of 0 °C and 10 °C and at a pressure of 34 atm.

In addition, the results from this study have been evaluated against various MOF-type materials as detailed in Table 5, including those reported by Sheng-Han et al. [86], Xiaoliang et al. [87], Bourrelly et al. [88], Zhang et al. [89], Zhou et al. [90], Zhao et al. [91], Millward et al. [92], and Furukawa et al. [93]. Notably, only three studies—Zhang et al. [89] (1007.6 mg·g^−1^), Millward et al. [92] (1493 mg·g^−1^), and Furukawa et al. [93] (2400 mg·g^−1^)—reported higher CO_2_ capture capacities at elevated pressures and 25 °C than the highest value from our study (827.8 mg·g^−1^ at 0 °C and 34 atm). However, these studies did not investigate adsorption–desorption cycles extensively (with Furukawa [93] conducting only a two-cycle study), which could impact the material’s structure and reduce its adsorption capacity. In contrast, our study involved multiple adsorption–desorption cycles, revealing a minor capacity loss of 1% to 1.5% after 10 cycles, underscoring its potential industrial application for purifying CO_2_-rich environments.

Moreover, the adsorption capacity was compared with adsorption capacities in previous studies. The obtained results for PMO–benzene at 34 atm and 0 °C (638.2 mg·g^−1^) was significantly higher than those of hydrotalcite Mg-Al (142.02 mg·g^−1^) [24] and organohydrotalcite (176.66 mg·g^−1^) [23], but was lower than the best result obtained with PMO–Ethane [61], which was 827.8 mg·g^−1^ at 0 °C and 34 atm.

Lastly, it is noteworthy to quantify the amount of adsorbent required to reduce current atmospheric CO_2_ levels (421 ppm (mL·m^−3^) [4]) to pre-industrial levels (280 ppm (mL·m^−3^) [1]). Given CO_2_’s density at 0 °C and 1 atm (1.976 mg·cm^−3^), the required amount would be 276.85 mg·m^−3^. Therefore, with a maximum capture capacity of 638.2 mg·g^−1^ at 0 °C, 0.43 g of PMO–benzene would suffice to lower the CO_2_ concentration in 1 m^3^ of air to pre-industrial levels. To apply this to a large volume, such as Wembley Stadium (1,139,100 m^3^), approximately 489.8 kg of PMO–benzene would be needed to achieve this reduction.

## 4. Conclusions

In this study, the adsorbent PMO–benzene was tested at high CO_2_ gas pressures (up to 35 atm) and low temperatures (0 °C, 10 °C, 20 °C, and 35 °C).

1. The pore size was between 2.4 and 4.1 nm, while the total pore volume was 0.68 cm^3^·g^−1^ and S_BET_ was 928 m^2^·g^−1^.
2. All isotherms fitted the Freundlich model well. nf > 1 was very close to 1, indicating favorable and weak interactions.
3. The Sips and Toth models improved the results obtained by other equations at temperatures between 0 °C and 20 °C, where multilayer formation occurred.
4. The D-R and Temkin models showed a physical nature of adsorption (E < 8 kJ·mol^−1^).
5. PMO–benzene featured the maximum adsorption (638.2 mg·g^−1^) at 0 °C and 34 atm.
6. These results highlight that 0.43 g of PMO–benzene would be enough to reduce the CO_2_ level in 1 m^3^ of air to pre-industrial levels; 489.8 kg of PMO–benzene would be required to reduce the CO_2_ concentration of the volume of Wembley soccer stadium to pre-industrial levels.
7. The maximum loss of adsorption capacity was 1.45% for the sample at 0 °C, after 10 adsorption–desorption cycles. Consequently, this material could be used in capture processes using changes in the pressure conditions.
8. PMO–benzene could contribute to the development of CO_2_ capture and use (CCU) technology.

## Figures and Tables

**Table 1:** Pore structure for PMO–benzene.

	S_BET_ (m^2^·g^−1^)	Smc ^1^ (m^2^·g^−1^)	Vp ^2^ (cm^3^·g^−1^)	Dp ^3^ (nm)
PMO–benzene	928	139	0.68	3.47

^1^ Micropore surface; ^2^ single-point pore volume; ^3^ average pore size diameter.

**Table 2:** Langmuir and Freundlich fitting results.

	Langmuir						Freundlich			
	qm	X_L_	qmc	K_L_	S_L_	R^2^	K_f_	n	nf	R^2^
	(mg·g^−1^)		(mg·g^−1^)	(atm^−1^)	(m^2^·g^−1^)		(mg·g^−1^·atm^(−1/n)^)		(1/n)	
PMO–benzene 0 °C	474.30	1.220	388.66	4.538	904.09	0.975	574.198	0.663	1.508	0.991
PMO–benzene 10 °C	429.93	1.218	353.01	4.590	821.18	0.982	505.880	0.643	1.556	0.998
PMO–benzene 20 °C	392.35	1.215	322.88	4.648	751.08	0.990	479.124	0.641	1.559	0.997
PMO–benzene 35 °C	357.14	1.238	288.57	4.208	671.27	0.997	468.582	0.692	1.445	0.996

**Table 3:** Sips and Toth model fitting results.

	Sips				Toth			
	q_S_	K_S_	n_S_	R^2^	q_T_	K_T_	n_T_	R^2^
	(mg·g^−1^)	(atm^−1^)			(mg·g^−1^)	(atm^−1^)		
PMO–benzene 0 °C	375.89	2.631	0.780	0.999	349.58	4.837	0.690	0.996
PMO–benzene 10 °C	384.79	1.981	0.780	1.000	357.72	4.303	0.760	0.999
PMO–benzene 20 °C	426.79	0.709	0.760	1.000	436.59	0.999	0.310	1.000
PMO–benzene 35 °C	-	-	-	-	-	-	-	-

**Table 4:** Dubinin–R and Temkin fitting results.

	Dubinin–Raduskevich				Temkin			
	q_D_	β	E	R^2^	K_Tk_	B	b_Tk_	R^2^
	(mg·g^−1^)	(mol^2^·kJ^−2^)	(kJ·mol^−1^)		(atm^−1^)		(kJ·mol^−1^)	
PMO–benzene 0 °C	440.54	0.035	3.780	0.897	27.711	143.096	0.016	0.847
PMO–benzene 10 °C	391.48	0.032	3.973	0.926	30.895	120.895	0.019	0.900
PMO–benzene 20 °C	344.76	0.028	4.208	0.961	37.432	100.181	0.024	0.949
PMO–benzene 35 °C	290.23	0.025	4.478	0.975	42.538	81.639	0.031	0.959

**Table 5:** CO_2_ capture for different materials.

Adsorbent	T Isotherm (°C)	Pressure (atm)	Capacity Adsorption (mg·g^−1^)	Ref.
PMO–benzene	25	1	22	[50]
PMO–benzene modified	25	1	133.32	
PMO–benzene (A-LB)	25	1	77.44	[51]
PMO–benzene (A-LBEO)	25	1	69.52	
PMO–Ethane Np py	0 and 25	1	68.2 and 40.5	[52]
PMO–Ethane Np Etbipy	0 and 25	1	73.04 and 41.8	
PMO–Ethane Np iPrbipy	0 and 25	1	99.44 and 45.7	
PMO-–Ethane	0	1	62.48	[53]
PMO-UDF	0	≈1	52.8	[54]
CPMOs	0	1	96.36	[55]
MCM-41-modified	75	1	133	[56]
NH_2_-Ph-PMO	25	≈10	114.4	[57]
TiO_2_/Graphene	25	1	82.72	[20]
MgO	25	1	79.2	[82]
ZSM-5 Mesoporous	40	1	39.6	[83]
MSiNTs-PEI50	85	0.6	121	[84]
CeO_2_ Mesoporous	25	10	391.6	[21]
PEI50	75	1	138.16	[85]
MOF-Al	0	1	124.52	[86]
MIL-53 (BNHx)	0	1	198	[87]
MIL-47 (V)	31	20	506	[88]
MIL-101	25	30	1007.6	[89]
MIL-101 (Cr, Mg)	25	1	145.2	[90]
MOF-5	22.85	1	92.4	[91]
MOF-74	25	42	457	[92]
MOF-177	25	42	1493	
MOF-200	25	50	2400	[93]
HT-MgAl-CO_3_	0	≈35	142.02	[24]
Organohydrotalcite TDD	0	35	176.66	[23]
PMO–benzene	0	≈34	638.2	This work
	10	≈34	465.2	
	20	≈34	346.7	
	35	≈34	266.0	

## Data Availability

The raw data supporting the conclusions of this article will be made available by the authors on request.

## References

[1] Bui M., Adjiman C.S., Bardow A., Anthony E.J., Boston A., Brown S., Fennell P.S., Fuss S., Galindo A., Hackett L.A. (2018). Carbon Capture and Storage (CCS): The Way Forward. Energy Environ. Sci..

[2] U.S. Department of Commerce, National Oceanic and Atmospheric Administration. https://www.noaa.gov/news-release/carbon-dioxide-now-more-than-50-higher-than-pre-industrial-levels#:~:text=PriortotheIndustrialRevolutionatmosphereforthousandsofyears.

[3] Friedlingstein P., Jones M.W., O’Sullivan M., Andrew R.M., Bakker D.C.E., Hauck J., Le Quéré C., Peters G.P., Peters W., Pongratz J. (2022). Global Carbon Budget 2021. Earth Syst. Sci. Data.

[4] Scripps Institution of Oceanography, UC San Diego The Keeling Curve. https://keelingcurve.ucsd.edu.

[5] United Nations Paris Agreement. https://unfccc.int/sites/default/files/english_paris_agreement.pdf.

[6] Masson-Delmotte V., Zhai P., Pörtner H.-O., Roberts D.C., Skea J., Shukla P.R., Pirani A., Moufouma-Okia W., Péan C., Pidcock R., IPCC (2018). Global Warming of 1.5 °C. An IPCC Special Report on the Impacts of Global Warming of 1.5 °C above Pre-Industrial Levels and Related Global Greenhouse Gas Emission Pathways, in the Context of Strengthening the Global Response to the Threat of Climate Change.

[7] Zhang W., Yang Y., Li Y., Li F., Luo M. (2023). Recent Progress on Integrated CO_2_ Capture and Electrochemical Upgrading. Mater. Today Catal..

[8] Vega L.F., Bahamon D., Alkhatib I.I.I. (2024). Perspectives on Advancing Sustainable CO_2_ Conversion Processes: Trinomial Technology, Environment, and Economy. ACS Sustain. Chem. Eng..

[9] Baena-Moreno F.M., Rodríguez-Galán M., Vega F., Alonso-Fariñas B., Vilches Arenas L.F., Navarrete B. (2019). Carbon Capture and Utilization Technologies: A Literature Review and Recent Advances. Energy Sources Part A Recover. Util. Environ. Eff..

[10] Salih H.A., Alkhatib I.I.I., Zahra M.A., Vega L.F. (2023). Diamine Based Hybrid-Slurry System for Carbon Capture. J. CO_2_ Util..

[11] Cox M., Mokaya R. (2017). Ultra-High Surface Area Mesoporous Carbons for Colossal Pre Combustion CO_2_ Capture and Storage as Materials for Hydrogen Purification. Sustain. Energy Fuels.

[12] Zhu B., Huang J., Lu J., Zhao D., Lu L., Jin S., Zhou Q. (2017). Worm-like Hierarchical Porous Carbon Derived from Bio-Renewable Lignin with High CO_2_ Capture Capacity. Int. J. Electrochem. Sci..

[13] Chen C., Feng N., Guo Q., Li Z., Li X., Ding J., Wang L., Wan H., Guan G. (2018). Template-Directed Fabrication of MIL-101(Cr)/Mesoporous Silica Composite: Layer-Packed Structure and Enhanced Performance for CO_2_ Capture. J. Colloid Interface Sci..

[14] Kishor R., Ghoshal A.K. (2015). APTES Grafted Ordered Mesoporous Silica KIT-6 for CO_2_ Adsorption. Chem. Eng. J..

[15] Licciulli A., Notaro M., De Santis S., Terreni C., Kunjalukkal Padmanabhan S. (2017). CO_2_ Capture on Amine Impregnated Mesoporous Alumina-Silica Mixed Oxide Spheres. Fuel Process. Technol..

[16] Wang X., Li H., Hou X.J. (2012). Amine-Functionalized Metal Organic Framework as a Highly Selective Adsorbent for CO_2_ over CO. J. Phys. Chem. C.

[17] Ding M., Flaig R.W., Jiang H.-L., Yaghi O.M. (2019). Carbon Capture and Conversion Using Metal–Organic Frameworks and MOF-Based Materials. Chem. Soc. Rev..

[18] Nandi S., De Luna P., Daff T.D., Rother J., Liu M., Buchanan W., Hawari A.I., Woo T.K., Vaidhyanathan R. (2015). A Single-Ligand Ultra-Microporous MOF for Precombustion CO_2_ Capture and Hydrogen Purification. Sci. Adv..

[19] Ho K., Jin S., Zhong M., Vu A.T., Lee C.H. (2017). Sorption Capacity and Stability of Mesoporous Magnesium Oxide in Post-Combustion CO_2_ Capture. Mater. Chem. Phys..

[20] Chowdhury S., Parshetti G.K., Balasubramanian R. (2015). Post-Combustion CO_2_ Capture Using Mesoporous TiO_2_/Graphene Oxide Nanocomposites. Chem. Eng. J..

[21] Liu G., Tatsuda K., Yoneyama Y., Tsubaki N. (2017). Synthesis of Mesoporous Cerium Compound for CO_2_ Capture. E3S Web Conf..

[22] Quesada Carballo L., Perez Perez M.d.R., Cantador Fernández D., Caballero Amores A., Fernández Rodríguez J.M. (2019). Optimum Particle Size of Treated Calcites for CO_2_ Capture in a Power Plant. Materials.

[23] Cantador Fernandez D., Suescum Morales D., Jiménez J.R., Fernández-Rodriguez J.M. (2022). CO_2_ Adsorption by Organohydrotalcites at Low Temperatures and High Pressure. Chem. Eng. J..

[24] Suescum-Morales D., Cantador-Fernández D., Jiménez J.R., Fernández J.M. (2021). Mitigation of CO_2_ Emissions by Hydrotalcites of Mg_3_Al-CO_3_ at 0 °C and High Pressure. Appl. Clay Sci..

[25] Karimi B., Ganji N., Pourshiani O., Thiel W.R. (2022). Periodic Mesoporous Organosilicas (PMOs): From Synthesis Strategies to Applications. Prog. Mater. Sci..

[26] Yang Q., Liu J., Zhang L., Li C. (2009). Functionalized Periodic Mesoporous Organosilicas for Catalysis. J. Mater. Chem..

[27] Esquivel D., De Canck E., Jimenez-Sanchidrian C., Van Der Voort P., Romero-Salguero F.J. (2014). Periodic Mesoporous Organosilicas as Catalysts for Organic Reactions. Curr. Org. Chem..

[28] Huang Y., Yuan P., Wu Z., Yuan X. (2016). Preparation of Surface-Silylated and Benzene-Bridged Ti-Containing Mesoporous Silica for Cyclohexene Epoxidation. J. Porous Mater..

[29] Horiuchi Y., Do Van D., Yonezawa Y., Saito M., Dohshi S., Kim T.-H., Matsuoka M. (2015). Synthesis and Bifunctional Catalysis of Metal Nanoparticle-Loaded Periodic Mesoporous Organosilicas Modified with Amino Groups. RSC Adv..

[30] López M., Esquivel D., Jiménez-Sanchidrián C., Romero-Salguero F., Van Der Voort P. (2015). A “one-step” Sulfonic Acid PMO as a Recyclable Acid Catalyst. J. Catal..

[31] Jiang Y., Liu X., Chen Y., Zhou L., He Y., Ma L., Gao J. (2014). Pickering Emulsion Stabilized by Lipase-Containing Periodic Mesoporous Organosilica Particles: A Robust Biocatalyst System for Biodiesel Production. Bioresour. Technol..

[32] Imamoglu M., Pérez-Quintanilla D., Sierra I. (2016). Bifunctional Periodic Mesoporous Organosilicas with Sulfide Bridges as Effective Sorbents for Hg(II) Extraction from Environmental and Drinking Waters. Microporous Mesoporous Mater..

[33] Esquivel D., Ouwehand J., Meledina M., Turner S., Van Tendeloo G., Romero-Salguero F.J., Clercq J.D., Voort P. (2017). Van Der Thiol-Ethylene Bridged PMO: A High Capacity Regenerable Mercury Adsorbent via Intrapore Mercury Thiolate Crystal Formation. J. Hazard. Mater..

[34] Ganiyu S.O., Bispo C., Bion N., Ferreira P., Batonneau-Gener I. (2014). Periodic Mesoporous Organosilicas as Adsorbents for the Organic Pollutants Removal in Aqueous Phase. Microporous Mesoporous Mater..

[35] Deka J.R., Liu C.L., Wang T.H., Chang W.C., Kao H.M. (2014). Synthesis of Highly Phosphonic Acid Functionalized Benzene-Bridged Periodic Mesoporous Organosilicas for Use as Efficient Dye Adsorbents. J. Hazard. Mater..

[36] Otero R., Esquivel D., Ulibarri M.A., Jiménez-Sanchidrián C., Romero-Salguero F.J., Fernández J.M. (2013). Adsorption of the Herbicide S-Metolachlor on Periodic Mesoporous Organosilicas. Chem. Eng. J..

[37] Lourenço M.A.O., Silva R.M., Silva R.F., Pinna N., Pronier S., Pires J., Gomes J.R.B., Pinto M.L., Ferreira P. (2015). Turning Periodic Mesoporous Organosilicas Selective to CO_2_/CH_4_ Separation: Deposition of Aluminium Oxide by Atomic Layer Deposition. J. Mater. Chem. A.

[38] Wu L., Yu Z., Ye Y., Yang Y., Zeng H., Huang J., Huang Y., Zhang Z., Xiang S. (2018). Sulfonated Periodic-Mesoporous-Organosilicas Column for Selective Separation of C_2_H_2_/CH_4_ Mixtures. J. Solid State Chem..

[39] Sanchez C., Jeremias F., Ernst S.J., Henninger S.K. (2017). Synthesis, Functionalization and Evaluation of Ethylene-Bridged PMOs as Adsorbents for Sorption Dehumidification and Cooling Systems. Microporous Mesoporous Mater..

[40] Lu D., Chen H., Yan X., Wang L., Zhang J. (2015). Ratiometric Hg^2+^ Sensor Based on Periodic Mesoporous Organosilica Nanoparticles and Förster Resonance Energy Transfer. J. Photochem. Photobiol. A Chem..

[41] Qiu X., Han S., Hu Y., Gao M., Wang H. (2014). Periodic Mesoporous Organosilicas for Ultra-High Selective Copper(Ii) Detection and Sensing Mechanism. J. Mater. Chem. A.

[42] Grösch L., Lee Y.J., Hoffmann F., Fröba M. (2015). Light-Harvesting Three-Chromophore Systems Based on Biphenyl-Bridged Periodic Mesoporous Organosilica. Chem. Eur. J..

[43] Zhou Z., Taylor R.N.K., Kullmann S., Bao H., Hartmann M. (2011). Mesoporous Organosilicas With Large Cage-Like Pores for High Efficiency Immobilization of Enzymes. Adv. Mater..

[44] Aggad D., Jimenez C.M., Dib S., Croissant J.G., Lichon L., Laurencin D., Richeter S., Maynadier M., Alsaiari S.K., Boufatit M. (2018). Gemcitabine Delivery and Photodynamic Therapy in Cancer Cells via Porphyrin-Ethylene-Based Periodic Mesoporous Organosilica Nanoparticles. ChemNanoMat.

[45] Inagaki S., Guan S., Fukushima Y., Ohsuna T., Terasaki O. (1999). Novel Mesoporous Materials with a Uniform Distribution of Organic Groups and Inorganic Oxide in Their Frameworks. J. Am. Chem. Soc..

[46] Melde B.J., Holland B.T., Blanford C.F., Stein A. (1999). Mesoporous Sieves with Unified Hybrid Inorganic/Organic Frameworks. Chem. Mater..

[47] Asefa T., MacLachlan M.J., Coombs N., Ozin G.A. (1999). Periodic Mesoporous Organosilicas with Organic Groups inside the Channel Walls. Nature.

[48] Kuroki M., Asefa T., Whitnal W., Kruk M., Yoshina-Ishii C., Jaroniec M., Ozin G.A. (2002). Synthesis and Properties of 1,3,5-Benzene Periodic Mesoporous Organosilica (PMO): Novel Aromatic PMO with Three Point Attachments and Unique Thermal Transformations. J. Am. Chem. Soc..

[49] Esquivel Merino M.D. (2011). Síntesis, Caracterización y Aplicaciones de Materiales Periódicos Mesoporosos Organosilícicos. Ph.D. Thesis.

[50] Sim K., Lee N., Kim J., Cho E.B., Gunathilake C., Jaroniec M. (2015). CO_2_ Adsorption on Amine-Functionalized Periodic Mesoporous Benzenesilicas. ACS Appl. Mater. Interfaces.

[51] Sim S., Cho E.-B. (2019). Multifunctional Periodic Mesoporous Benzene-Silicas for Evaluation of CO_2_ Adsorption at Standard Temperature and Pressure. Microporous Mesoporous Mater..

[52] Kirren P., Barka L., Rahmani S., Bondon N., Donzel N., Trens P., Bessière A., Raehm L., Charnay C., Durand J.O. (2022). Periodic Mesoporous Organosilica Nanoparticles for CO_2_ Adsorption at Standard Temperature and Pressure. Molecules.

[53] Wei Y., Li X., Zhang R., Liu Y., Wang W., Ling Y., El-Toni A.M., Zhao D. (2016). Periodic Mesoporous Organosilica Nanocubes with Ultrahigh Surface Areas for Efficient CO_2_ Adsorption. Sci. Rep..

[54] Liu M., Lu X., Shi L., Wang F., Sun J. (2017). Periodic Mesoporous Organosilica with a Basic Urea-Derived Framework for Enhanced Carbon Dioxide Capture and Conversion Under Mild Conditions. ChemSusChem.

[55] Rekha P., Sharma V., Mohanty P. (2016). Synthesis of Cyclophosphazene Bridged Mesoporous Organosilicas for CO_2_ Capture and Cr (VI) Removal. Microporous Mesoporous Mater..

[56] Xu X., Song C., Andresen J.M., Miller B.G., Scaroni A.W. (2002). Novel Polyethylenimine-Modified Mesoporous Molecular Sieve of MCM-41 Type as High-Capacity Adsorbent for CO_2_ Capture. Energy Fuels.

[57] Lourenço M.A.O., Siquet C., Sardo M., Mafra L., Pires J., Jorge M., Pinto M.L., Ferreira P., Gomes J.R.B. (2016). Interaction of CO_2_ and CH_4_ with Functionalized Periodic Mesoporous Phenylene-Silica: Periodic DFT Calculations and Gas Adsorption Measurements. J. Phys. Chem. C.

[58] Burleigh M.C., Markowitz M.A., Jayasundera S., Spector M.S., Thomas C.W., Gaber B.P. (2003). Mechanical and Hydrothermal Stabilities of Aged Periodic Mesoporous Organosilicas. J. Phys. Chem. B.

[59] Burleigh M.C., Jayasundera S., Thomas C.W., Spector M.S., Markowitz M.A., Gaber B.P. (2004). A Versatile Synthetic Approach to Periodic Mesoporous Organosilicas. Colloid Polym. Sci..

[60] International Centre for Diffraction Data (ICDD) (2003). The Powder Diffraction.

[61] Cantador-Fernandez D., Suescum-Morales D., Esquivel D., Jiménez J.R., Fernández-Rodriguez J.M. (2023). CO_2_ Adsorption by Ethane Periodic Mesoporous Organosilica at Low Temperatures and High Pressure. J. Environ. Chem. Eng..

[62] Langmuir I. (1916). The Constitution and Fundamental Properties of Solids and Liquids. Part I. Solids. J. Am. Chem. Soc..

[63] Langmuir I. (1918). The Adsorption of Gases on Plane Surfaces of Glass, Mica and Platinum. J. Am. Chem. Soc..

[64] Freundlich H. (1907). Über Die Adsorption in Lösungen. Z. Phys. Chem..

[65] Sips R. (1948). On the Structure of a Catalyst Surface. J. Chem. Phys..

[66] Tóth J. (1995). Uniform Interpretation of Gas/Solid Adsorption. Adv. Colloid Interface Sci..

[67] Dubinin M.M. (1985). Generalization of the Theory of Volume Filling of Micropores to Nonhomogeneous Microporous Structures. Carbon.

[68] Dubinin M.M., Zaverina E.D. (1949). Sorbtsiya I Struktura Aktivnykh Uglei. 6. Strukturnye Tipy Aktivnykh Uglei. J. Phys. Chem..

[69] Temkin M., Pyzhev V. (1940). Kinetics of Ammonia Synthesis on Promoted Iron Catalyst. Acta Phys. Chim. USSR.

[70] Temkin M., Levich V. (1946). Adsorption Equilibrium on Heterogeneous Surfaces. J. Phys. Chem..

[71] Liang Y., Liu X., Allen M.R. (2018). Measuring and Modeling Surface Sorption Dynamics of Organophosphate Flame Retardants on Impervious Surfaces. Chemosphere.

[72] Limousin G., Gaudet J.-P., Charlet L., Szenknect S., Barthes V., Krimissa M. (2007). Sorption Isotherms: A Review on Physical Bases, Modeling and Measurement. Appl. Geochem..

[73] Hu Q., Wang Q., Feng C., Zhang Z., Lei Z., Shimizu K. (2018). Insights into Mathematical Characteristics of Adsorption Models and Physical Meaning of Corresponding Parameters. J. Mol. Liq..

[74] Sparks D.L. (2003). Environmental Soil Chemistry.

[75] Saadi R., Saadi Z., Fazaeli R., Fard N.E. (2015). Monolayer and Multilayer Adsorption Isotherm Models for Sorption from Aqueous Media. Korean J. Chem. Eng..

[76] Lee H.I., Pak C., Yi S.H., Shon J.K., Kim S.S., So B.G., Chang H., Yie J.E., Kwon Y.-U., Kim J.M. (2005). Systematic Phase Control of Periodic Mesoporous Organosilicas Using Gemini Surfactants. J. Mater. Chem..

[77] Haul R.S.J., Gregg K.S.W. (1982). Sing: Adsorption, Surface Area and Porosity.

[78] Sing K.S.W., Everett D.H., Haul R.A.W., Moscou L., Pierotti R.A., Rouquerol J., Siemieniewska T. (2008). Reporting Physisorption Data for Gas/Solid Systems. Handbook of Heterogeneous Catalysis.

[79] Thommes M., Kaneko K., Neimark A.V., Olivier J.P., Rodriguez-Reinoso F., Rouquerol J., Sing K.S.W. (2015). Physisorption of Gases, with Special Reference to the Evaluation of Surface Area and Pore Size Distribution (IUPAC Technical Report). Pure Appl. Chem..

[80] Giles C.H., MacEwan T.H., Nakhwa S.N., Smith D. (1960). 786. Studies in Adsorption. Part XI. A System of Classification of Solution Adsorption Isotherms, and Its Use in Diagnosis of Adsorption Mechanisms and in Measurement of Specific Surface Areas of Solids. J. Chem. Soc..

[81] Esfandiari B., Monajjemi M. (2013). Physical Adsorption between Mono and Diatomic Gases inside of Carbon Nanotube with Respect to Potential Energy. J. Phys. Theor. Chem..

[82] Bhagiyalakshmi M., Lee J.Y., Jang H.T. (2010). Synthesis of Mesoporous Magnesium Oxide: Its Application to CO_2_ Chemisorption. Int. J. Greenh. Gas Control.

[83] Wang Y., Du T., Song Y., Che S., Fang X., Zhou L. (2017). Amine-Functionalized Mesoporous ZSM-5 Zeolite Adsorbents for Carbon Dioxide Capture. Solid State Sci..

[84] Niu M., Yang H., Zhang X., Wang Y., Tang A. (2016). Amine-Impregnated Mesoporous Silica Nanotube as an Emerging Nanocomposite for CO_2_ Capture. ACS Appl. Mater. Interfaces.

[85] Li K., Jiang J., Tian S., Yan F., Chen X. (2015). Polyethyleneimine–Nano Silica Composites: A Low-Cost and Promising Adsorbent for CO_2_ Capture. J. Mater. Chem. A.

[86] Lo S.-H., Chien C.-H., Lai Y.-L., Yang C.-C., Lee J.J., Raja D.S., Lin C.-H. (2013). A Mesoporous Aluminium Metal–Organic Framework with 3 Nm Open Pores. J. Mater. Chem. A.

[87] Si X., Zhang J., Li F., Jiao C., Wang S., Liu S., Li Z., Zhou H., Sun L., Xu F. (2012). Adjustable Structure Transition and Improved Gases (H_2_, CO_2_) Adsorption Property of Metal–Organic Framework MIL-53 by Encapsulation of BNHx. Dalt. Trans..

[88] Bourrelly S., Llewellyn P.L., Serre C., Millange F., Loiseau T., Férey G. (2005). Different Adsorption Behaviors of Methane and Carbon Dioxide in the Isotypic Nanoporous Metal Terephthalates MIL-53 and MIL-47. J. Am. Chem. Soc..

[89] Zhang Z., Huang S., Xian S., Xi H., Li Z. (2011). Adsorption Equilibrium and Kinetics of CO_2_ on Chromium Terephthalate MIL-101. Energy Fuels.

[90] Zhou Z., Mei L., Ma C., Xu F., Xiao J., Xia Q., Li Z. (2016). A Novel Bimetallic MIL-101(Cr, Mg) with High CO_2_ Adsorption Capacity and CO_2_/N_2_ Selectivity. Chem. Eng. Sci..

[91] Zhao Z., Li Z., Lin Y.S. (2009). Adsorption and Diffusion of Carbon Dioxide on Metal−Organic Framework (MOF-5). Ind. Eng. Chem. Res..

[92] Millward A.R., Yaghi O.M. (2005). Metal-Organic Frameworks with Exceptionally High Capacity for Storage of Carbon Dioxide at Room Temperature. J. Am. Chem. Soc..

[93] Furukawa H., Ko N., Go Y.B., Aratani N., Choi S.B., Choi E., Yazaydin A.O., Snurr R.Q., O’Keeffe M., Kim J. (2010). Ultrahigh Porosity in Metal-Organic Frameworks. Science.

